# Antimicrobial Drug Resistance of *Salmonella* Isolates from Meat and Humans, Denmark

**DOI:** 10.3201/eid1304.060748

**Published:** 2007-04

**Authors:** Marianne N. Skov, Jens Strodl Andersen, Søren Aabo, Steen Ethelberg, Frank M. Aarestrup, Anders Hay Sørensen, Gitte Sørensen, Karl Pedersen, Steen Nordentoft, Katharina E.P. Olsen, Peter Gerner-Smidt, Dorte L. Baggesen

**Affiliations:** * University of Southern Denmark, Odense, Denmark; † Danish Institute for Food and Veterinary Research, Copenhagen, Denmark; ‡ Statens Serum Institut, Copenhagen, Denmark

**Keywords:** Salmonella, zoonoses, multidrug resistance, antimicrobial drug resistance, meat products, imported meat, humans, dispatch

## Abstract

We compared 8,144 *Salmonella* isolates collected from meat imported to or produced in Denmark, as well as from Danish patients. Isolates from imported meat showed a higher rate of antimicrobial drug resistance, including multidrug resistance, than did isolates from domestic meat. Isolates from humans showed resistance rates lower than those found in imported meat but higher than in domestic meat. These findings indicate that programs for controlling resistant *Salmonella* spp. are a global issue.

*Salmonella* spp. are among the most common causes of human bacterial gastroenteritis worldwide, and food animals are important reservoirs of the bacteria (*1*). In recent years, an increase in the occurrence of antimicrobial drug–resistant *Salmonella* spp. has been observed in several countries (*2**–**5*). Fatality rates are higher for patients with infections caused by drug-resistant *Salmonella* spp., and these patients are more likely to require hospitalization and to be hospitalized for longer periods than are patients with infections caused by antimicrobial drug–susceptible *Salmonella* spp. (*6**,**7*).

Antimicrobial drug resistance of *Salmonella* spp. isolated from food animals in Denmark has so far been relatively low (*8*). However, an estimated 30% of all poultry, 10% of all pork, and 50% of all beef sold in Denmark is imported (*9*). Imported meat is therefore an important potential source of human infection with drug-resistant *Salmonella* spp. We compared antimicrobial drug resistance of *Salmonella* isolates from both imported meat and meat produced within Denmark (domestic meat), as well as from outpatients with diarrhea.

*Salmonella* isolates from humans and meat were obtained from July 1998 through June 2002. Isolates from domestic poultry, pork, and beef were obtained through the national *Salmonella* control program (*10*), and isolates from imported poultry, pork, and beef were obtained from the Denmark import control and from the regional food control units. Human salmonellosis is a notifiable disease in Denmark, and all human *Salmonella* spp. isolates are collected at the Statens Serum Institute. The serovars included were restricted to *S.* Typhimurium, *S.* Hadar, *S.* Dublin, *S.* Saintpaul*, S.* Enteritidis, *S.* Virchow, and *S.* Newport because these were the serovars of which a sufficient number of isolates had been tested for antimicrobial drug susceptibility. Data on 4,081 *Salmonella* isolates from humans were included in the study.

Identification, serotyping, phage typing, and susceptibility testing were done as described (*8**,**11**,**12*). Susceptibility to the following antimicrobial agents was determined: ampicillin, ceftiofur, chloramphenicol, ciprofloxacin, co-amoxiclav, colistin, florphenicol, gentamicin, nalidixic acid, neomycin, streptomycin, sulfamethoxazole, tetracycline, and trimethoprim.

Statistical analyses were performed using S-PLUS version 6.2 (Insightful Corp., Seattle, WA, USA). The trend in the occurrence of resistant isolates over time, the occurrence of multidrug-resistant isolates over time, and the occurrence of nalidixic acid–resistant isolates were investigated by fitting a logistic regression model with origin (domestic/imported), time (year), product type (beef, pork, poultry), and all 2-way interactions as explanatory variables. The regression models were reduced by using a likelihood ratio test. Significance in all 2-by-2 tables (only tables with minimum 30 domestic and 30 imported samples) was tested by a Pearson χ^2^ test with continuity correction; if the number in any cell in the contingency table was <5, Fisher exact test was applied. All tests were done on a 5% significance level (p<0.05**)**. No correction for multiple testing was done. An isolate was considered multidrug resistant if the isolate was resistant to >4 antimicrobial agents.

*Salmonella* spp. were isolated from 1,078 (11.8%) of 9,135 samples from imported poultry, pork, and beef and 2,985 (1.4%) of 213,214 samples from domestic poultry, pork, and beef. Among the isolates from domestic meat, the serovars *S*. Typhimurium, *S*. Infantis, and *S*. Derby were the 3 most frequently isolated; in imported meat, the 3 most frequently isolated serovars were *S*. Heidelberg, *S*. Typhimurium, and *S*. Hadar (Table 1). In isolates from domestic meat originating from pigs and poultry, *S*. Typhimurium was the most frequently isolated serovar; in beef isolates, *S*. Dublin was most common. Among isolates from imported meat, *S*. Typhimurium was the most frequently isolated serovar from pork and beef, while *S*. Heidelberg was the most frequently isolated serovar from poultry.

**Table 1:** Number and proportion of susceptible (S), resistant (R), multidrug-resistant (M), and nalidixic acid–resistant (Nal) *Salmonella* spp. isolates within different serotypes isolated from meat and humans, Denmark, July 1998–July 2002*

Serotype	Domestic meat					Imported meat					Humans†				
	No. tested	%				No. tested	%				No. tested	%			
		S	R	M	Nal		S	R	M	Nal		S	R	M	Nal
Typhimurium‡	1,508	73	21	6	1	138	34	24	42§	9§	1,886	61	20	19	3
Infantis‡	184	94	4	2	2	50	84	10	6	8§					
Derby‡	163	55	44	1	1	34	32	59	9§	3					
Heidelberg	6	67	33	0	17	157	49	13	38	4					
Hadar‡	38	74	26	0	11	113	1	53§	46§	81§	189	26	71	3	58
Enteritidis‡	91	90	9	1	4	50	84	16	0	10	1,706	92	7	0	4
Indiana‡	94	95	4	1	0	40	45	43§	13§	3					
Newport	2	0	100	0	100	78	28	51	21	60	59	88	7	5	5
Kottbus	26	81	19	0	15	49	6	90	4	92					
Dublin	71	99	1	0	1	4	100	0	0	0	88	95	5	0	2
Anatum	50	100	0	0	0	12	75	8	17	0					
Saintpaul	9	11	0	89	22	39	31	8	62	15	58	72	9	19	7
Regent	47	0	100	0	100	1	0	100	0	0					
Virchow	3	100	0	0	0	39	44	36	21	49	95	35	56	9	62
Bredeney	3	100	0	0	0	38	34	0	66	11					
Other‡	690	71	24	5	5	256	56	24	20	17					
Total	2,985	74	22	4	4	1,078	42	30	28	26	4,081	73	17	9	7

*Only serotypes with ≥40 isolates are included.  †Only subsets of selected serovar are routinely tested for susceptibility to antimicrobial agents. ‡Indicates serotypes with >30 samples from Danish produced meat and >30 samples from imported meat, which were statistically tested.  §Indicates clinical significance.

A significantly higher (χ^2^, p<0.001) proportion of the *Salmonella* spp. isolates from imported meat (58%) were resistant to ≥1 antimicrobial agents compared with isolates from domestic meat (26%) (Table 1). A significant difference (χ^2^, p<0.001) was also observed between the proportions of multidrug-resistant isolates from domestic (4%) compared with imported (28%) poultry, pork, and beef.

The regression results (Table 2) showed a significant increase in the proportion of resistant (p<0.001) and multidrug-resistant (p = 0.015) isolates over time and an increase in odds per year of 27% (corresponding to an increase in probability of 5% per year) and 14% (corresponding to an increase in probability of 3% per year), respectively (Figure 1). Furthermore, the probability for isolating a resistant and a multidrug-resistant isolate from imported meat compared with domestic meat was significant, with an odds ratio of ≈5. The probability of isolating a resistant isolate differed between product types; pork had the highest probability, followed by poultry and beef.

**Table 2:** Results from the reduced logistic regression models*

Variable	OR (95% CI)	Estimate (95% CI)	SE (Est.)	p value
Resistance vs. nonresistance				
Intercept	0.164 (0.129 to 0.207)	–1.81 (–2.05 to –1.57)	0.121	
Origin	5.08 (4.19 to 6.18)	1.62 (1.43 to 1.82)	0.0988	<0.00001
Year	1.27 (1.19 to 1.35)	0.235 (0.174 to 0.297)	0.0313	<0.00001
Cattle vs. poultry	0.400 (0.230 to 0.662)	–0.917 (–1.47 to –0.413)	0.268	<0.00001
Pork vs poultry	1.26 (1.06 to 1.51)	0.233 (0.0553 to 0.414)	0.0916	
Multidrug resistance vs. resistance				
Intercept μ	0.141 (0.0976 to 0.201)	–1.96 (–2.33 to –1.60)	0.185	
Origin	4.98 (3.87 to 6.44)	1.61 (1.35 to 1.86)	0.129	<0.00001
Year	1.14 (1.03 to 1.27)	0.133 (0.0259 to 0.240)	0.0547	0.0148
Nalidixic acid resistance vs. non–nalidixic acid resistance				
Intercept	0.0611 (0.0333 to 0.107)	–2.80 (–3.40 to –2.24)	0.296	
Origin	6.54 (3.45 to 12.8)	1.88 (1.24 to 2.55)	0.334	
Year	1.41 (1.18 to 1.69)	0.342 (0.167 to 0.526)	0.0914	
Origin and year	0.732 (0.587 to 0.909)	–0.311 (–0.532 to –0.0956)	0.111	0.00448
Cattle vs. poultry	0.0404 (0.00229 to 0.182)	–3.21 (–6.08 to –1.70)	1.01	<0.00001
Pork vs. poultry	0.0668 (0.0425 to 0.101)	–2.71 (–3.16 to –2.29)	0.220	

*OR, odds ratio; CI, confidence interval; SE, **standard error; Est., estimated.**

**Figure 1:**
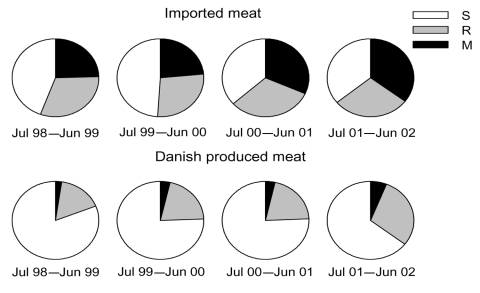
Proportion of susceptible (S), resistant (R), and multidrug-resistant (M) *Salmonella* isolates from domestic and imported meat, Denmark, July 1998–July 2002.

A high proportion of resistant and multidrug-resistant isolates was found among *S*. Hadar, *S*. Newport, *S*. Typhimurium, and *S.* Heidelberg in imported meat (Table 1). Among *S*. Typhimurium, antimicrobial drug resistance was particularly prominent in the phage types DT104, DT170, DT193, DT120, DT208, DT107, U302, and DT135 (Table 3). Multidrug-resistant DT104, DT120, and DT193 were found in both domestic and imported poultry, pork, and beef, whereas multidrug-resistant DT107, DT170, and DT208 were more common in domestic meat, and multidrug-resistant U302 was more common in imported meat (Table 3).

**Table 3:** Number and proportion of susceptible (S), resistant (R), and multidrug-resistant (M) meat isolates within *Salmonella* Typhimurium phage types, Denmark, July 1998–July 2002*

Serovar/phage type	Domestic meat				Imported meat				Total no.
	M, %	R, %	S, %	Total no.	M, %	R, %	S, %	Total no.	
All *S.* Typhimurium isolates	6	21	73	1,508	42	24	34	138	1,646
DT104	70	13	17	23	88	7	5	43	66
DT170	3	68	29	97	0	0	0	0	97
DT193	13	37	51	63	50	17	33	6	69
DT120	16	29	55	38	57	43	0	7	45
DT208	57	40	3	30	0	57	43	7	37
DT107	5	55	41	22	0	0	0	0	22
U302	0	33	67	6	38	31	31	13	19
DT135	6	56	38	16	0	100	0	2	18
Other *S.* Typhimurium	5	21	74	1,213	21	32	47	60	1,273
*Salmonella* minus Typhimurium	3	22	75	1,477	26	31	43	940	2,417
Total	4	22	74	2,985	28	30	42	1,078	4,063

*Not all *S.* Typhimurium isolates from humans were phage typed.

Resistance to nalidixic acid was higher among isolates from imported meat (26%) compared with isolates from domestic meat (4%) (χ^2^, p<0.001, odds ratio = 6.54, Table 3), with an increase over time in the proportion of domestic nalidixic acid–resistant isolates (p = 0.004, data not shown). Furthermore, the probability of isolating a nalidixic acid–resistant isolate differed between product types; poultry (domestic 14%, imported 30%) had the highest probability, followed by pork (domestic 1%, imported 3.2%) and beef (domestic 1%, imported 0%). Nalidixic acid resistance among *Salmonella* spp. from imported products was highest among *S*. Hadar, *S*. Newport, *S*. Kottbus, and *S*. Virchow (Table 1).

For *S*. Typhimurium, *S*. Hadar, and *S*. Virchow, the proportion of resistant and multidrug-resistant isolates was much higher among isolates from humans than among isolates from domestic meat (Table 1, Figure 2). For *S*. Dublin and *S.* Enteritidis, the proportion of resistant and multidrug-resistant isolate did not differ between the meat sources and the human isolates, whereas for *S*. Saintpaul and *S.* Newport the rates of resistance and multidrug resistance were lower for isolates from humans than from both domestic and imported meat.

**Figure 2:**
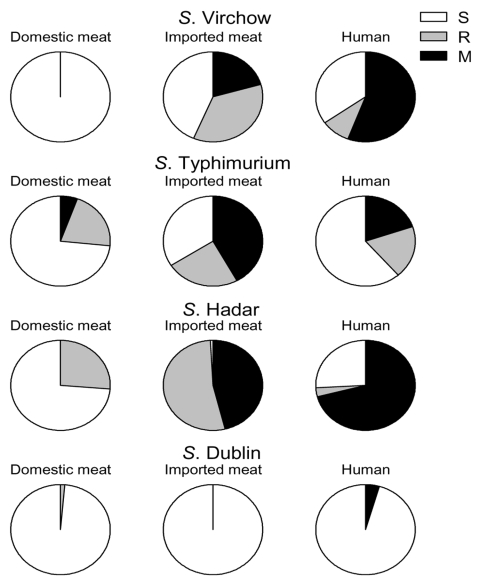
Proportion of susceptible (S), resistant (R), and multidrug-resistant (M) isolates among different *Salmonella* serotypes in isolates from domestic meat, imported meat, and humans, Denmark, July 1998–July 2002.

*S.* Hadar, *S.* Virchow, *S.* Newport, and *S.* Heidelberg were frequently found in imported products but rarely found in domestic products. Isolates that belong to these serovars are common causes of human salmonellosis in Denmark (*13*). Overall, a significantly higher number of resistant and multidrug-resistant *Salmonella* isolates were found among isolates from imported poultry, pork, and beef compared with domestic products. This finding implies that consumers in Denmark are more likely to be exposed to drug-resistant *Salmonella* spp. when eating imported compared with domestic meat. An increase in the occurrence of resistance over time was also observed among isolates from both domestic and imported meat; this is in agreement with observations worldwide (*2**–**5*). Antimicrobial agents might not be essential for treatment of gastroenteritis caused by *Salmonella* spp., but they are essential for treatment of patients with invasive infections. In particular, the frequent occurrence of resistance to quinolones is a matter of concern because these compounds are often used for first treatment of serious human infections, before the results of susceptibility testing are available.

International trade of food products is expected to increase in the future. Thus, endeavors to improve food safety must take into account the importance of resistant *Salmonella* spp. in imported food products and, through international agreements, limit contamination with antimicrobial drug–resistant *Salmonella* spp. at the primary production site.
